# Knowledge, Attitudes, and Safety Practices About COVID-19 Among High School Students in Iran During the First Wave of the Pandemic

**DOI:** 10.3389/fpubh.2021.680514

**Published:** 2021-08-04

**Authors:** Hossein Hatami, Mohsen Abbasi-Kangevari, Mohammad-Reza Malekpour, Ali-Asghar Kolahi

**Affiliations:** ^1^Department of Public Health, School of Public Health and Safety and Environmental and Occupational Hazards Control Research Center, Shahid Beheshti University of Medical Sciences, Tehran, Iran; ^2^Social Determinants of Health Research Center, Shahid Beheshti University of Medical Sciences, Tehran, Iran

**Keywords:** attitudes, coronavirus, knowledge, perceptions, safety practices, SARS-CoV-2, COVID-19

## Abstract

**Background:** School closures have inevitably deprived students of their traditional source of information. The objective of this study was to determine knowledge, attitudes, and safety practices about COVID-19 among high school students in Iran.

**Methods:** This study was conducted from March 24th-April 3rd, 2020. Data were collected via an online-parent-administrative questionnaire.

**Results:** Responses of 704 students were analyzed. Students' mean (SD) knowledge score was 21.5 (4.6) of 30. More than 90% of students knew about the cause of the disease, the routes of transmission, and the most renowned symptoms: dyspnea and cough. Social-and- audiovisual-media were the leading information source. Most students believed that people need to keep safe physical distancing, everyone should isolate themselves upon symptoms onset, people should avoid unnecessary in-person contact with family and friends, and that cities need to go under lockdowns if needed. Students' mean (SD) practice score was 20.2 (2.5) of 24. Most students did not go on a trip, and more than 80% said they would wear facemasks when going outside.

**Conclusions:** High school students' knowledge and safety practices about COVID-19 were somewhat satisfactory, and their attitudes toward the disease were mainly positive. Nevertheless, some witnessed knowledge gaps, negative attitudes, and unsafe practices in the study highlighted the need for targeted education on the pandemic. Social and mass media's significant role and potential could be utilized to battle misinformation and deliver proper knowledge to young adolescents.

## Background

The coronavirus disease 2019 (COVID-19) pandemic has resulted in more than 180 million reported cases and 3.9 million deaths worldwide (1). Most governments had temporarily closed all schools during the first wave of the pandemic to contain the spread of the disease, affecting over 1.5 billion children or 90% of the world's student population (2). Children do not seem to be the direct victims of the adverse health effects of COVID-19. However, the pandemic has the potential to deeply affect young children's development worldwide profoundly, especially their mental and physical well-being, the short-term and long-term repercussions of which are yet to be understood (3, 4).

Schools have been closed due to the COVID-19 pandemic in Iran. School closures inevitably increased the use of online learning platforms (3). The deprivation of students of their traditional source of information has increased their risk to online threats such as the spread of misinformation primarily via social media, which could fuel the potential misunderstandings (5). Accurate information about the disease and the current situation could prevent irrational worries and concerns of children and minimize their anxiety or fear (6). There have been reports of negative stigmatizing attitudes and irresponsible safety practices toward COVID-19. Higher knowledge regarding COVID−19 is associated with a lower likelihood of negative attitudes and potentially dangerous practices (7). Safe health practices and adherence to controlling measures are especially essential and could determine the future course of the pandemic (8).

As an early step toward facilitating the outbreak management and ensuring safe practices, high school students' educational needs need to be addressed to provide training programs via the appropriate media by public health authorities. Therefore, current knowledge, attitudes, and safety practices need to be rapidly investigated, especially in the early days of school closures due to lockdown in Iran. The objective of this study was to determine the knowledge, attitudes, and safety practices of the high school students in Iran about COVID-19 via a rapid online survey administered by students' parents.

## Methods

This study was approved by the Ethical Committee of Shahid Beheshti University of Medical Sciences under code IR.SBMU.RETECH.REC.1399.412. Participation was anonymous and upon students' and parental consent.

### Setting and Sampling

This study was conducted from March 24th to April 3rd, 2020, in Iran. Data were collected via an online parent-administrative questionnaire in the Google Docs platform. The questionnaire's link was posted on popular social networks in Iran, including Telegram, WhatsApp, Instagram, and Twitter. The questionnaire instructed that parents of high school students read the questions to their children and fill in the questionnaire on their behalf strictly based on their answers. It was emphasized that parents do not change the answers so that we could have a better understanding of their children's educational needs about COVID-19. Participants were high school students, grades 6–12, who lived in the 31 provinces of Iran. Participants who were studying in primary school or were not currently living in Iran were excluded from the study.

### Variables and the Questionnaire

Variables included sociodemographic characteristics, knowledge about COVID-19, attitudes toward COVID-19, and safety practices during the pandemic. Sociodemographic characteristics included students' age, sex, ethnicity, high school grade, knowing someone with COVID-19, and the province of residence in Iran.

The questionnaire was in plain Persian and consisted of four main sections: sociodemographic characteristics, knowledge, attitudes, and safety practices about COVID-19.

The knowledge section assessed students' knowledge about cause, transmission, symptoms, red flags, prevention, treatment, sensitive groups, and follow-up for COVID-19; studens' self-evaluation on their level of knowledge, their source of information, and their felt need for further training.

The attitudes section included statements about participants' attitudes toward COVID-19. In response to these statements, participants chose an item from the five-point Likert scale.

The safety practices section assessed students' self-isolation, care-seeking, preventive measures, and going to public places during the pandemic.

A panel of experts evaluated the content validity of the questionnaire. An item discrimination analysis was conducted for each scale to eliminate too tricky or easy items. Factor analysis was performed for factor structure. A separate test-retest over 2 weeks was held for the three scales of the questionnaire. The test-retest correlation for the knowledge scale was 0.89; Kuder-Richardson-20 was used to prevent the overestimation of internal consistency; the coefficient was 0.83. The test-retest correlation for the attitudes scale was 0.91; the coefficient alpha was 0.87. The test-retest correlation for the practice scale was 0.87; the coefficient alpha was 0.88. The pilot survey was conducted on fifty female and fifty male students. The English and Persian versions of the questionnaire are available in Supplementary Table 1.

### Data Analysis

One point was awarded to each correct answer to score students' knowledge about COVID-19. To analyze students' attitudes, “I strongly agree” and “I agree” were considered as “I agree”; and “I strongly disagree” and “I disagree” were considered as “I disagree.” To analyze students' safety practices, one point was awarded to taking each right measure or not taking each wrong measure. The 95% confidence interval (95% CI) was reported for the key proportions using the exact binomial distribution. Categorical variables were analyzed by the Chi-Square test. For analyzing the differences among means of two groups and three groups or more, independent-sample *t*-test and one-way analysis of variance (ANOVA) test were used. Statistical analyses were performed using IBM SPSS Statistics 21. A probability level of <0.05 was considered significant. Figures were designed by Tableau Desktop 2018 (Washington, USA).

## Results

The responses of 704 students were analyzed. The mean (SD) age of students was 16.5 (1.7) [range = 10–19, being 16.6 (1.7) among girls, and 16.4 (1.6) among boys]. As many as 371 (52.7%) participants reported they knew someone with COVID-19. Other sociodemographic characteristics of students are presented in Table 1.

**Table 1:** Sociodemographic characteristics of participants.

**Variable**	**N (%)**
Sex	
Female	397 (56.4)
Male	307 (43.6)
Ethnicity	
Fars	389 (55.3)
Turk/Azari	132 (18.8)
Kurd	45 (6.4)
Lor	38 (5.4)
Other	100 (14.1)
High school grade	
First 3 years	153 (21.7)
Second 3 years	551 (78.3)

### Knowledge

#### Symptoms and Red Flags of COVID-19

More than 90% of students recognized that COVID-19 could present itself with dyspnea and cough. Although some 98% knew that worsening dyspnea could be a red flag, some 68% considered persistent fever dangerous. As many as 26 (3.8%) and 33 (4.6%) reported they did not know what the symptoms of and red flags of severe disease were, respectively (Table 2, Supplementary Figures 1, 2).

**Table 2:** Students' knowledge on symptoms of COVID-19 and red-flags of severe disease.

**Symptoms/Red flags**	**Yes (%)**	**No (%)**
**Symptoms (** ***n*** **=** **678)**		
Dyspnea	635 (93.7)	43 (6.3)
Fever	627 (92.5)	51 (7.5)
Cough	595 (87.8)	83 (12.2)
Loss of smell or taste	427 (63.0)	251 (37.0)
Sore throat	314 (46.3)	364 (53.7)
Malaise	313 (46.2)	365 (53.8)
Myalgia	286 (42.2)	392 (57.8)
Diarrhea	241 (35.5)	437 (64.5)
Sneeze	171 (25.2)	507 (74.8)
Loss of appetite	159 (23.5)	519 (76.5)
Rhinorrhea	120 (17.7)	558 (82.3)
**Red flags (** ***n*** **=** **671)**		
Worsening dyspnea	655 (97.6)	16 (2.4)
Worsening coughs	476 (70.9)	195 (29.1)
Fever > 5 days	454 (67.7)	217 (32.3)
Loss of consciousness	105 (15.6)	566 (80.4)
Confusion	62 (9.2)	609 (90.8)

#### General Knowledge About COVID-19

More than 90% of students correctly recognized the cause of COVID-19, its routes of transmission, and the necessity of self-isolation (Table 3). The mean (SD) knowledge score was 21.5 (4.6), range = 1–30, which is 71.6% of 30. No significant differences were observed between students' knowledge scores and their age, sex, province, knowing someone with COVID-19, having a household member with COVID-19, having the disease, or having a family member passing away from COVID-19.

**Table 3:** Students' general knowledge about COVID-19.

**Statement**	**It's true (%)**	**It's false (%)**	**I don't know (%)**
An infected travel companion could transmit COVID-19.	684 (97.2)	9 (1.3)	11 (1.6)
COVID-19 could be transmitted via infected respiratory droplets and surfaces.	683 (97.0)	5 (0.7)	16 (2.3)
Patients with COVID-19 and their close contacts should be quarantined >14 days.	660 (93.8)	6 (0.9)	38 (5.4)
The cause of COVID-19 is a virus.	567 (93.3)	13 (1.8)	34 (4.8)
Solely washing hands with water is not enough for disinfection.	555 (78.8)	105 (14.9)	44 (6.3)
No specific treatment is currently available for COVID-19.	607 (86.2)	29 (4.1)	68 (9.7)
There are still no medications available for COVID-19 prevention.	575 (81.7)	52 (7.4)	77 (10.9)
Solely using the hand dryer would not kill the cause of COVID-19.	462 (65.6)	106 (15.1)	136 (19.3)
Vaccination against pneumonia or influenza has no protection against COVID-19.	420 (59.7)	63 (8.9)	221 (31.4)
Eating garlic does not kill the cause of COVID-19.	400 (56.8)	112 (15.9)	192 (27.3)
Insects cannot transmit COVID-19 to humans.	312 (44.3)	104 (14.8)	288 (40.9)
Regularly rinsing nostrils with saline has a protective effect against COVID-19	278 (39.5)	210 (29.8)	216 (30.7)
Mouth washing has protective effect against COVID-19.	210 (29.8)	251 (35.7)	243 (34.5)
Hand rubbing with alcohol-based solutions is more effective than handwashing with soap.	203 (28.8)	416 (59.1)	85 (12.1)
Air humidifiers could kill the cause of COVID-19.	182 (25.9)	336 (47.7)	186 (26.4)
COVID-19 only causes mild symptoms.	176 (25.0)	380 (54.0)	148 (21.0)
All patients with COVID-19 need to be admitted in the hospital.	78 (11.1)	582 (82.7)	44 (6.3)
People recovered from COVID-19 will never get the disease again.	73 (10.4)	443 (62.9)	188 (26.7)
Children do not get COVID-19.	63 (8.9)	587 (83.4)	54 (7.7)
A person with no cough, fever, or dyspnea cannot carry the pathogen.	60 (8.5)	602 (85.5)	42 (6.0)
Patients with COVID-19 who do not have a fever cannot transmit the disease.	59 (8.4)	545 (77.4)	100 (14.2)
Taking a shower with hot water can kill the agent inside the body.	59 (8.4)	523 (74.3)	122 (17.3)
COVID-19 has always a severity like the common cold.	58 (8.2)	572 (81.3)	74 (10.5)
Warming upper airways with a blow dryer can kill the pathogen inside the body.	37 (5.3)	583 (82.8)	84 (11.9)
The influenza virus causes COVID-19.	30 (4.3)	561 (79.7)	113 (16.1)
Going to a steam room or sauna can kill the pathogen inside the body.	28 (4.0)	547 (77.7)	129 (18.3)
Drinking alcohol beverages could kill the pathogen inside the body.	27 (3.8)	613 (87.1)	64 (9.1)
A person who recovered from COVID-19 does not need to follow prevention protocols.	5 (0.7)	684 (97.2)	15 (2.1)

#### Students' Self-Evaluation on Their Level of Knowledge About COVID-19

As many as 203 (28.8) students considered their level of knowledge sufficient, 450 (63.9) fair, and 51 (7.2) insufficient. Subsequently, when asked about their felt need for further training, 429 (60.9) said yes. As indicated in Table 4, audiovisual media and Telegram were the leading sources of information about COVID-19 among students, and online school classes had less contribution in this regard (Supplementary Figure 3).

**Table 4:** Students' sources of information about COVID-19.

**Source of information**	***n* (%)**
Audiovisual media	537 (76.3)
Telegram	528 (75.0)
Searching the internet	392 (55.7)
Family and friends	366 (52.0)
Instagram	290 (41.2)
News agencies	248 (35.2)
Healthcare professionals	236 (33.5)
Online school classes	87 (12.4)
Print media	66 (9.4)
Pamphlets and posters	60 (8.5)
Twitter	53 (7.5)

### Attitudes

When asked about the reaction if they had COVID-19, 644 (91.5%) reported seeing a doctor in case symptoms get worse, 614 (87.2%) self-isolation at home, while 14 (2.0%) continuing daily life.

As many as 165 (23.4%) said, it is very highly likely that COVID-19 is made for bioterrorism, 190 (27.0%) high, 203 (28.8%) fair, 80 (11.4%) low, and 66 (9.4%) very low.

About the students' evaluation on how dangerous the current situation is, 308 (43.8%) said very high, 299 (42.5%) high, 84 (11.9%) fair, 8 (1.1%) low, and 5 (0.7%) very low.

While 610 (86.6%) students thought that nations would finally defeat the disease, 94 (13.4%) said COVID-19 could not be defeated.

In response to a question asking how long it would take to control this pandemic, 7 (1.0%) said less than a month, 131 (18.6%) 1–3 months, 218 (31.0%) 3–6 months, 119 (16.9%) 6–9 months, 106 (15.1%) 9–12 months, 106 (15.1) more than a year, and 17 (2.3%) had no idea.

Most students had positive attitudes toward COVID-19. They believed that people need to keep safe physical distancing, everyone should isolate themselves upon symptoms onset, and that cities need to go under lockdowns if needed. However, more than 20% thought the pandemic is divine retribution for the sins of humankind, and they were most uncomfortable to be around a person who has recently recovered from COVID-19 (Table 5).

**Table 5:** Attitudes of participants toward COVID-19.

**Statement**	**Number of respondents**	**I agree (%)**	**I disagree (%)**
Keeping safe physical-distance is the duty of all people.	696	693 (99.6)	3 (0.4)
I would visit a friend or family member with COVID-19.	695	7 (1.0)	688 (99.0)
Only people with predisposing factors need to take preventive measures.	682	27 (4.0)	655 (96.0)
School closure is an opportunity to visit family and friends.	670	48 (7.2)	622 (92.8)
Every household member should use their towel or facial tissue to dry hands and face.	669	653 (97.6)	16 (2.4)
I would notify school authorities upon the emergence of COVID-19 symptoms.	659	652 (98.9)	7 (1.1)
All affected cities need to go under immediate lockdown.	631	595 (94.3)	36 (5.7)
Relatives of a person deceased from COVID-19 should not feel ashamed.	605	580 (95.9)	25 (4.1)
Pet owners should take special care of their pets' contacts with people.	605	566 (93.6)	39 (6.4)
People with a positive travel history or close contacts should report themselves to health authorities.	601	573 (95.3)	28 (4.7)
Authorities need to prioritize more critical issues like road-accidents.	599	35 (5.8)	564 (94.2)
I would rather shop online instead of going shopping in person.	584	550 (94.2)	34 (5.8)
Despite keeping a safe distance, I feel uncomfortable around people recovered from COVID-19.	516	323 (62.6)	193 (37.4)
This pandemic is divine retribution due to the sins of humankind.	466	98 (21.0)	368 (79.0)
The pandemic resolves on its own when the weather gets warmer.	456	77 (16.9)	379 (83.1)

### Safety Practices

#### Self-Isolation and Care-Seeking

Among students, 3 (0.4) reported they had become COVID-19 positive, 603 (85.7) reported they did not get the disease, and 98 (13.9) reported they had some symptoms but took no measures.

#### Preventive Measures for COVID-19

When asked about what they did to prevent COVID-19, 644 (91.5) students reported staying at home, 638 (90.6) practicing hand hygiene, 470 (66.8) keeping safe physical distance, 517 (73.4) wearing gloves, and 512 (72.7) wearing a facemask, 238 (33.8) taking vitamin supplements, 164 (23.3) drinking herbal tea, 13 (1.8) taking Imam-Kazem-drug, 5 (0.7) performing wet cupping.

#### Wearing Facemasks

Among 446 students, 177 (39.7) reported they wear surgical masks, 148 (33.2) cloth masks, 154 (34.5) N95 masks, and 57 (12.8) homemade masks. However, 50 (11.2) respondents reported they did not wear masks. The mean (SD) duration of waring each mask was 5.3 (8.7) h, median (IQR) = 3.0 (2–5).

#### Leaving the House

The mean (SD) times of going outside the house during the past week were 1.8 (3.2), median (IQR) = 1 (0–2). About the reason to leave the house, 269 (38.2) reported grocery shopping, 44 (6.3) shopping items other than groceries, 69 (9.8) visiting family and friends, 67 (9.5) sightseeing, and 19 (2.7) going to healthcare facilities. As many as 254 (36.1) reported they did not leave the house. Among male students, 78 (25.4%) stated they did not leave the house during the pandemic, which was 176 (44.3%) among female participants, *p* < 0.001.

#### Handwashing

The mean (SD) times of daily handwashing with water and soap among students was 9.0 (9.9), median (IQR) = 6 (4–10). The mean (SD) duration of handwashing was 23.4 (16.8) seconds, median (IQR) = 20 (15–30).

The mean (SD) times of daily hand-rub with alcohol-based hand sanitizers among students was 3.8 (9.3), median (IQR) = 2 (0–4). The mean (SD) duration of hand-rub was 9.2 (9.9) seconds, median (IQR) = 10 (0–15).

#### General Safety Practices About COVID-19

The mean (SD) score for general safety practices about COVID-19 was 20.2 (2.5), range = 4–24, which is 84.1% of 24 (Table 6). No significant differences were observed between practice scores of students and their age, sex, province of residence, whether they knew someone with COVID-19, whether they had a household member with COVID-19, whether they caught the disease, or whether a family member had passed away from COVID-19.

**Table 6:** General statements regarding safety practices of students about COVID-19.

**Statement**	**Yes (%)**	**No (%)**
I will not go on a trip during Nowruz.	686 (97.4)	18 (2.6)
I will isolate myself at home if I become symptomatic.	661 (93.9)	43 (6.1)
As requested by authorities, I stayed at home and isolated myself immediately.	650 (92.3)	54 (7.7)
I disinfect the packaging of the goods I buy from stores.	646 (91.8)	58 (8.2)
I did not go on a trip during Nowruz.	646 (91.8)	58 (8.2)
I cover my mouth and nose when sneezing or coughing.	616 (87.5)	88 (12.5)
I always use a facemask when going outside the house.	581 (82.5)	123 (17.5)
I bought some unprescribed drugs to prevent the disease.	575 (81.7)	129 (18.3)
I always disinfect my hands before touching my face.	568 (80.7)	136 (19.3)
I carry hand-sanitizers or water-soap solutions outside the home.	539 (76.6)	165 (23.4)
I regularly disinfect my cellphone according to guidelines.	533 (75.7)	171 (24.3)
I bought some facemasks for personal protection.	523 (74.3)	181 (25.7)
I heat the bread before use to kill pathogens.	491 (69.7)	213 (30.3)
I regularly disinfect highly-touched surfaces at home.	484 (68.7)	220 (31.3)
I keep my cellphone in my pocket to reduce the chance of infection.	472 (67.0)	232 (33.0)
I made homemade facemasks for me/my family.	132 (18.8)	572 (81.3)
I went to Bazaar for shopping on days before Nowruz.	77 (10.9)	627 (89.1)
As always, I visited family and friends during Nowruz.	18 (2.6)	686 (97.4)
I attended a relative's funeral.	15 (2.1)	689 (97.9)
I visited a graveyard on the last Thursday before Nowruz.	10 (1.4)	694 (98.6)
As always, I will visit family and friends during Nowruz.	10 (1.4)	694 (98.6)

## Discussion

Students' knowledge and safety practices about COVID-19 were somewhat adequate, and their attitudes toward the disease were positive. Most students knew that dyspnea and cough could be symptoms of COVID-19. In comparison, only half of the young adults in Karachi in a study recognized fever as a disease symptom (9). Almost all students recognized respiratory droplets as a transmission route, similar to a study among senior pharmacy students in Egypt (10). The majority of students said there were no medications available for the disease at the time, which was higher than the undergraduate students in a study in Italy (11). There were misbeliefs about COVID-19 among students. Although hand hygiene with soap and water is strongly recommended for disease prevention (12), some students said that washing hands without soap is sufficient for disinfection. Furthermore, some said that using hand dryers could effectively kill the pathogen, which is considered a myth (13).

Some 40% of students were unsure if COVID-19 could be transmitted via insects. There was no evidence of COVID-19 transmission by blood-feeding arthropods such as mosquitoes at the study time (14). However, it is now known that SARS-CoV-2 fails to infect or replicate in mosquitoes (15). The witnessed level of uncertainty regarding transmission routes calls for education campaigns by education and healthcare authorities.

While Telegram and the audiovisual media were the sources of information for most students, only 12% mentioned online school classes as their source of information about COVID-19. No studies were available which investigated the usage pattern of social media among Iranian adolescents. Our results illustrated the lack of school engagement in increasing students' awareness regarding different aspects of COVID-19. It is worth mentioning that the lockdown could have provided a more significant opportunity to watch television and surf the internet while staying at home (16). Mass media are valuable resources for efficiently forming people's knowledge and attitudes, improving their risk perception, and adequately informing people about risks and precautions (17). Although social media has played a significant role in information dissemination, its contribution to disseminating misinformation is noticeable (18). Social media provides a unique platform for engagement with experts. However, the lack of scientific peer review can contribute to mass hysteria rather than alleviating it. Since filtering the knowledge from the misinformation is hugely challenging in social media, the focus must be on reliable sources which avoid personal opinion or conjecture (19). This calls for extensive collaboration among public health authorities and the media outlets to provide and deliver specific health information to all the sectors of the society, especially children and young adolescents whose level of contribution to COVID-19 transmission on a community level has remained controversial (20).

There were no significant differences among students' knowledge score and their province of residence. This could suggest that the information origin of the popular media was the same, led by the national audiovisual media. Although the gender of the students was not a determinant of their level of knowledge, there are studies where women were more knowledgeable about COVID-19 (7, 11). No significant differences were found in the knowledge scores of students of various ethnicities. On the contrary, a study in the United States reported significant differences between white people and other ethnicities (21).

Most students believed that nations would finally defeat the disease, which is higher than a study among senior pharmacy students in Egypt (10) and similar to a study among the public in Malaysia (22). The majority of children expected that the pandemic would be controlled within a year, highlighting their hopes and belief in the scientific community and healthcare authorities. In the meantime, most students believed that people need to follow safe practices as suggested by healthcare authorities.

The pandemic unlocks some potentials of currently available technology and thus transforms people's behavior (23). Consumers are adapting to movement restrictions and are adopting newer technologies to facilitate work. More and more people are choosing online shopping to avoid unnecessary transport and contact with others. In our study, 94% of students reported that they would rather shop online instead of shopping in person.

Some 36% of students said they did not leave their houses during the preceding week, which is significant, especially considering that the early days of movement restrictions coincided with the holidays for the Persian new year, Nowruz, which are at least 2 weeks. Iranians have rituals before and during Nowruz. Before Nowruz, they go shopping for new clothes, shoes, sweets, and nuts, which are crucial for children and young adolescents. They also visit graveyards for deceased beloved ones on the last Thursday before Nowruz. During the holidays, Iranians visit family and friends or go on trips. However, some 90% of students reported that they did not go to the Bazaar before Nowruz and did not go on trips during the holidays. Students had a relatively safe hygiene practice. Only 11% of students said they did not wear masks while going outside, which is much lower than the 50% among senior pharmacy students in a study in Egypt (10) or the public in Malaysia (22). Nevertheless, some students followed traditional methods to prevent the disease. This could raise concerns, as the use of unapproved medications could result in unknown consequences (24).

## Strengths and Limitations

This is a rapid online survey to assess the knowledge, attitudes, and safety practices of high school students in Iran about COVID-19. Although in the recent infectious outbreaks, some concerns about Iranian students' knowledge, attitudes, and safety practices were raised (25), a study that specifically considers high school students has not been conducted in previous infectious outbreaks in Iran. The strengths are its rather large sample and data gathering in the early days of the outbreak in Iran. Findings could empower education and public health authorities better to understand the current educational needs of high school students to be addressed. We realize the limitations of the study. Due to the complexity of some questions in the questionnaire of the present study, only high school students were included in the study. Nevertheless, the knowledge, attitudes, and safety practices of primary school students need to be investigated, considering that they are also quite influenced by social media (26). The administration of the study questionnaire via parents of the students could result in some impairments in the reflection of truths. Although we had explicitly asked parents not to change their children's answers, such voluntary measures are not guaranteed. Since there was no representative online platform to connect researchers to people in Iran, we decided to conduct this study in the Google Forms platform. The penetration rate of the Internet in Iran is high. However, some groups, especially those in rural areas, might not have access to the platform.

## Conclusions

High school students' knowledge and safety practices about COVID-19 were somewhat satisfactory, and their attitudes toward the disease were mainly positive. Nevertheless, some witnessed knowledge gaps, negative attitudes, and unsafe practices in the study highlighted the need for targeted education on the pandemic. The significant role and potential of social and mass media could be utilized to battle misinformation and deliver proper knowledge to young adolescents.

## Data Availability Statement

The data that support the findings of this study are available from Social Determinants of Health Research Center, Shahid Beheshti University of Medical Sciences, Tehran, Iran but restrictions apply to the availability of these data, which were used under license for the current study, and so are not publicly available. Data are however available from the authors upon reasonable request and with permission of Shahid Beheshti University of Medical Sciences, Tehran, Iran.

## Ethics Statement

This study was approved by the Ethical Committee of Shahid Beheshti University of Medical Sciences under code IR.SBMU.RETECH.REC.1399.412. Participation was anonymous and upon the participant's own decision and parental consent. Written informed consent to participate in this study was provided by the participants' legal guardian/next of kin.

## Author Contributions

A-AK, HH, and MA-K: conceptualization. A-AK, MA-K, and M-RM: data collection. MA-K and M-RM: data analysis. HH and MA-K: writing—original draft. A-AK, MA-K, and HH: writing—review and editing. A-AK: resources and supervision. All authors have read and approved the manuscript prior to submission.

## Conflict of Interest

The authors declare that the research was conducted in the absence of any commercial or financial relationships that could be construed as a potential conflict of interest.

## Publisher's Note

All claims expressed in this article are solely those of the authors and do not necessarily represent those of their affiliated organizations, or those of the publisher, the editors and the reviewers. Any product that may be evaluated in this article, or claim that may be made by its manufacturer, is not guaranteed or endorsed by the publisher.

## Supplementary Material

Supplementary Figure 1 Students' knowledge on symptoms of COVID-19.

Supplementary Figure 2 Students' knowledge on red-flags of severe disease.

Supplementary Figure 3 Students' sources of information about COVID-19.

Supplementary Table 1 The English and Persian versions of the study questionnaire.

## Abbreviations

COVID-19: The coronavirus disease 2019
ANOVA: One-way analysis of variance
SD: Standard deviation.
