# Supplementary figures and images for: Knowledge, Attitudes, and Safety Practices About COVID-19 Among High School Students in Iran During the First Wave of the Pandemic
Source: Front Public Health. 2021 Aug 4;9:680514. doi: 10.3389/fpubh.2021.680514 (PMC8371395; doi:10.3389/fpubh.2021.680514)

# Symptoms

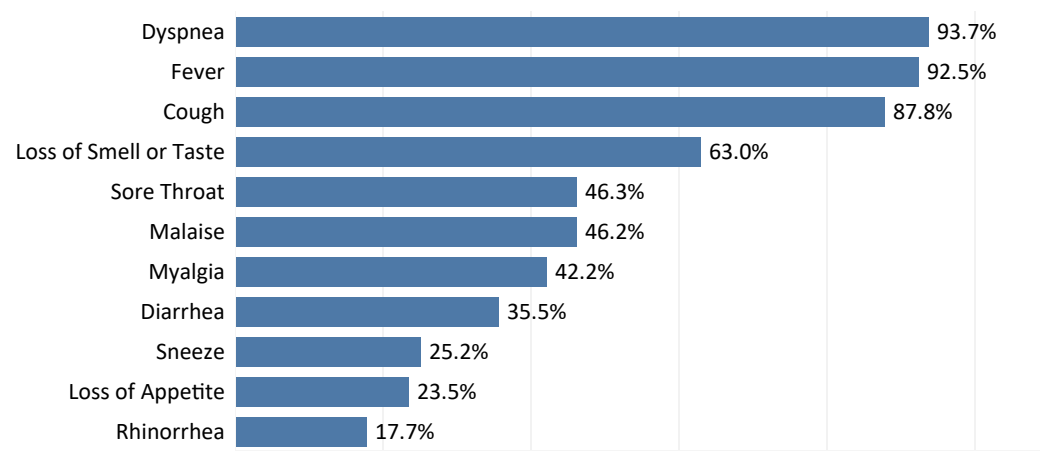

Supplement: Supplementary Figure 1 — Students' knowledge on symptoms of COVID-19. [file Data_Sheet_1.PDF]

Red Flags

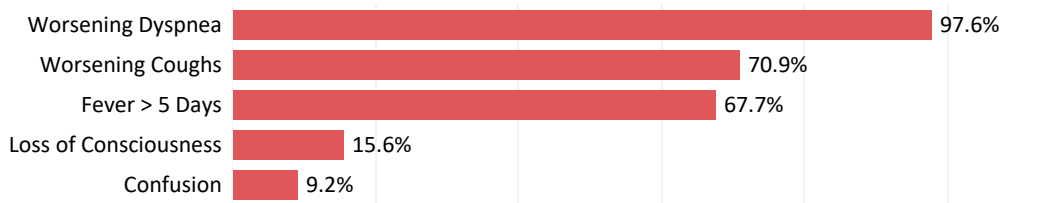

Supplement: Supplementary Figure 2 — Students' knowledge on red-flags of severe disease. [file Data_Sheet_2.PDF]

## Students' sources of Information about COVID-19

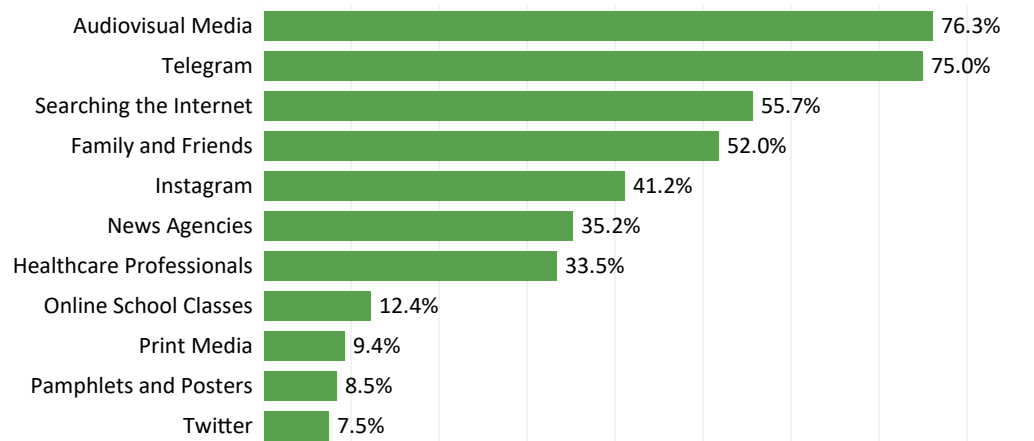

Supplement: Supplementary Figure 3 — Students' sources of information about COVID-19. [file Data_Sheet_3.PDF]
