# Supplementary material for: Knowledge, Attitudes, and Safety Practices About COVID-19 Among High School Students in Iran During the First Wave of the Pandemic
Source: Front Public Health. 2021 Aug 4;9:680514. doi: 10.3389/fpubh.2021.680514 (PMC8371395; doi:10.3389/fpubh.2021.680514)
Supplement: Supplementary Table 1 — The English and Persian versions of the study questionnaire. [file Data_Sheet_4.PDF]

# Shahid Beheshti University of Medical Sciences

## Knowledge, attitudes, and practices about COVID-19 among high school students in Iran

| | | | |
|---|---|---|---|
| C1 | Participant code: | C2 | Time stamp: |
| C3 | Province of residence: | C4 | City of residence: |

Dear parents, this questionnaire has been developed by Shahid Beheshti University of Medical Sciences to assess your child’s knowledge, attitudes, and practices about COVID-19. If your child wishes to participate in the study, and if you consent, it would be appreciated if you would take time to read the questions aloud to your child and submit their exact answers. Please kindly note that your sound report of your child’s answers would help us have a better understanding of their educational needs during the pandemic. Be assured that all data will remain confidential and that you could leave the study at any stage.

| | |
|---|---|
| C5 | Age: ….. years |
| C6 | Sex: Female □ Male □ |
| C7 | Ethnicity: Azari/Turk □ Fars □ Kurd □ Lor □ Other (please specify) □ ……. |
| C8 | High school grade: First three years □ Second three years □ |
| C9 | Do you know anyone who has/has had COVID-19? Yes □ No □ |

**Which of the following statements are true in your opinion?**

**(True □ False □ I don’t know □)**

| | |
|---|---|
| K1.1 | Solely using the hand dryer would not kill the cause of COVID-19. |
| K1.2 | Vaccination against pneumonia or influenza has no protection against COVID-19. |
| K1.3 | COVID-19 only causes mild symptoms. |
| K1.4 | Air humidifiers could kill the cause of COVID-19. |
| K1.5 | Taking a shower with hot water can kill the agent inside the body. |
| K1.6 | Going to a steam room or sauna can kill the pathogen inside the body. |
| K1.7 | Warming upper airways with a blow dryer can kill the pathogen inside the body. |
| K1.8 | COVID-19 has always a severity like the common cold. |
| K1.9 | All patients with COVID-19 need to be admitted in the hospital. |
| K1.10 | Hand rubbing with alcohol-based solutions is more effective than handwashing with soap. |
| K1.11 | Patients with COVID-19 who do not have a fever cannot transmit the disease. |
| K1.12 | Eating garlic does not kill the cause of COVID-19. |
| K1.13 | There are still no medications available for COVID-19 prevention. |
| K1.14 | No specific treatment is currently available for COVID-19. |
| K1.15 | A person with no cough, fever, or dyspnea cannot carry the pathogen. |
| K1.16 | Regularly rinsing nostrils with saline has a protective effect against COVID-19 |
| K1.17 | Mouth washing has protective effect against COVID-19. |
| K1.18 | Solely washing hands with water is not enough for disinfection. |
| K1.19 | The influenza virus causes COVID-19. |

| | |
|---|---|
| K1.20 | The cause of COVID-19 is a virus. |
| K1.21 | Insects cannot transmit COVID-19 to humans. |
| K1.22 | COVID-19 could be transmitted via infected respiratory droplets and surfaces. |
| K1.23 | Patients with COVID-19 and their close contacts should be quarantined >14 days. |
| K1.24 | A person who recovered from COVID-19 does not need to follow prevention protocols. |
| K1.25 | Children do not get COVID-19. |
| K1.26 | People recovered from COVID-19 will never get the disease again. |
| K1.27 | An infected travel companion could transmit COVID-19. |

| | | | | |
|---|---|---|---|---|
| **Which of the following could a symptom of COVID-19?** | | | | |
| K2.1 | Loss of smell or taste | Yes □ | No □ | I don't know □ |
| K2.2 | Diarrhea | Yes □ | No □ | I don't know □ |
| K2.3 | Rhinorrhea | Yes □ | No □ | I don't know □ |
| K2.4 | Loss of appetite | Yes □ | No □ | I don't know □ |
| K2.5 | Fever | Yes □ | No □ | I don't know □ |
| K2.6 | Dyspnea | Yes □ | No □ | I don't know □ |
| K2.7 | Myalgia | Yes □ | No □ | I don't know □ |
| K2.8 | Cough | Yes □ | No □ | I don't know □ |
| K2.9 | Malaise | Yes □ | No □ | I don't know □ |
| K2.10 | Sneeze | Yes □ | No □ | I don't know □ |
| K2.11 | Sore throat | | | |
| K2.12 | Other (please specify) …………………………………………………………… | | | |
| **Which of the following could be a possible red-flag for COVID-19?** | | | | |
| K3.1 | Fever > 5 days | Yes □ | No □ | I don't know □ |
| K3.2 | Worsening dyspnea | Yes □ | No □ | I don't know □ |
| K3.3 | Worsening coughs | Yes □ | No □ | I don't know □ |
| K3.4 | Loss of consciousness | Yes □ | No □ | I don't know □ |
| K3.5 | Confusion | Yes □ | No □ | I don't know □ |
| K3.6 | Other (please specify) …………………………………………………………… | | | |
| T1 | **Do you think you have enough information about COVID-19?** Yes □ To some extent □ No □ | | | |
| T2 | **Which of the following is your source of information about COVID-19?** (More than one option could be chosen)<br>Audiovisual media □ Telegram □ Newspaper and magazines □<br>Posters/Brochures □ Healthcare professionals □ Online school classes □<br>Instagram □ Twitter □ Family/Friends □ Other (please specify) □ ......... | | | |
| T3 | **Do you think you need to be trained in COVID-19?** Yes □ No □ | | | |

| | |
|---|---|
| A1 | **What would you do if you got COVID-19?**<br>Seeing a doctor in case symptoms get worse □<br>Self-isolation at home □<br>Continuing daily life □ |
| A2 | **How likely is it that COVID-19 is made for bioterrorism?**<br>Very high □ High □ Fair □ Low □ Very low □ |
| A3 | **How dangerous do you consider the current situation?**<br>Very high □ High □ Fair □ Low □ Very low □ |
| A4 | **Do you think that nations would finally defeat the disease?**<br>Yes □ No □ |
| A5 | **How long do you think it would take to control this pandemic?** |

| | |
|---|---|
| | Less than a month □ 1-3 months □ 3-6 months □ 6-9 months □ 9-12 months □ More than a year □ No idea □ |
| **To what extend do you agree or disagree with the following statements?** | |
| A6.1 | I would visit a friend or family member with COVID-19.<br>I strongly agree □ I agree □ No opinion □ I disagree □ I strongly disagree □ |
| A6.2 | I would rather shop online instead of going shopping in person.<br>I strongly agree □ I agree □ No opinion □ I disagree □ I strongly disagree □ |
| A6.3 | Despite keeping a safe distance, I feel uncomfortable around people recovered from COVID-19.<br>I strongly agree □ I agree □ No opinion □ I disagree □ I strongly disagree □ |
| A6.4 | I would notify school authorities upon the emergence of COVID-19 symptoms.<br>I strongly agree □ I agree □ No opinion □ I disagree □ I strongly disagree □ |
| A6.5 | The pandemic resolves on its own when the weather gets warmer.<br>I strongly agree □ I agree □ No opinion □ I disagree □ I strongly disagree □ |
| A6.6 | Relatives of a person deceased from COVID-19 should not feel ashamed.<br>I strongly agree □ I agree □ No opinion □ I disagree □ I strongly disagree □ |
| A6.7 | Only people with predisposing factors need to take preventive measures.<br>I strongly agree □ I agree □ No opinion □ I disagree □ I strongly disagree □ |
| A6.8 | School closure is an opportunity to visit family and friends.<br>I strongly agree □ I agree □ No opinion □ I disagree □ I strongly disagree □ |
| A6.9 | Keeping safe physical-distance is the duty of all people.<br>I strongly agree □ I agree □ No opinion □ I disagree □ I strongly disagree □ |
| A6.10 | Every household member should use their towel or facial tissue to dry hands and face.<br>I strongly agree □ I agree □ No opinion □ I disagree □ I strongly disagree □ |
| A6.11 | Pet owners should take special care of their pets' contacts with people.<br>I strongly agree □ I agree □ No opinion □ I disagree □ I strongly disagree □ |
| A6.12 | Authorities need to prioritize more critical issues like road-accidents.<br>I strongly agree □ I agree □ No opinion □ I disagree □ I strongly disagree □ |
| A6.13 | People with a positive travel history or close contacts should report themselves to health authorities.<br>I strongly agree □ I agree □ No opinion □ I disagree □ I strongly disagree □ |
| A6.14 | All affected cities need to go under immediate lockdown.<br>I strongly agree □ I agree □ No opinion □ I disagree □ I strongly disagree □ |
| A6.15 | This pandemic is divine retribution due to the sins of humankind.<br>I strongly agree □ I agree □ No opinion □ I disagree □ I strongly disagree □ |

| | | |
|---|---|---|
| P1 | **Have you had COVID-19?** | Yes, I got COVID-19 positive □<br>No □<br>I had some symptoms, but did nothing □ |
| P2 | **What did you do to prevent COVID-19? (**More than one option could be chosen)<br>Staying at home □<br>Practicing hand hygiene □<br>Keeping safe physical distance □<br>Wearing gloves □<br>Wearing a facemask □<br>Taking vitamin supplements □<br>Drinking herbal tea □<br>Taking Imam-Kazem-drug □<br>Performing wet cupping □<br>Other (Please specify) □ ……………………… | |

| | |
|---|---|
| P3 | How long do you wear a mask before its disposal? |
| P4 | **What kind of mask do you use for COVID-19 prevention?**<br>I do not wear masks □<br>Surgical mask □<br>N95 mask □<br>Cloth mask □<br>Homemade mask □<br>Other (Please specify) □ ……………………… |
| P5 | How many times have you left the house in the previous week? |
| P6 | **Why did you leave the house?**<br>I did not leave the house in the previous week □<br>Grocery shopping □<br>Shopping items other than groceries □<br>Visiting family and friends □<br>Sightseeing □<br>Going to healthcare facilities □ |
| P7 | How many times do you wash your hands with water and soup daily? |
| P8 | How long do you wash your hands with water and soup? |
| P9 | How many times do you hand-rub with alcohol-based hand sanitizers daily? |
| P10 | How long do you hand-rub with alcohol-based hand sanitizers? |

| **Which of the following measures did you take during the pandemic?** | | |
|---|---|---|
| P11.1 | I bought some unprescribed drugs to prevent the disease. | □ |
| P11.2 | I bought some facemasks for personal protection. | □ |
| P11.3 | I made homemade facemasks for me/my family. | □ |
| P11.4 | I carry hand-sanitizers or water-soap solutions outside the home. | □ |
| P11.5 | I attended a relative's funeral. | □ |
| P11.6 | I visited a graveyard on the last Thursday before Nowruz. | □ |
| P11.7 | I went to Bazaar for shopping on days before Nowruz. | □ |
| P11.8 | I always disinfect my hands before touching my face. | □ |
| P11.9 | I heat the bread before use to kill pathogens. | □ |
| P11.10 | I regularly disinfect highly-touched surfaces at home. | □ |
| P11.11 | I keep my cellphone in my pocket to reduce the chance of infection. | □ |
| P11.12 | I regularly disinfect my cellphone according to guidelines. | □ |
| P11.13 | As always, I visited family and friends during Nowruz. | □ |
| P11.14 | As always, I will visit family and friends during Nowruz. | □ |
| P11.15 | I will isolate myself at home if I become symptomatic. | □ |
| P11.16 | As requested by authorities, I stayed at home and isolated myself immediately. | □ |
| P11.17 | I always use a facemask when going outside the house. | □ |
| P11.18 | I always disinfect my hands before touching my face. | □ |
| P11.19 | I cover my mouth and nose when sneezing or coughing. | □ |
| P11.20 | I did not go on a trip during Nowruz. | □ |
| P11.21 | I will not go on a trip during Nowruz. | □ |

دانشگاه علوم‌پزشکی شهید بهشتی

**پرسشنامه بررسی آگاهی، نگرش و عملکرد دانش‌آموزان دبیرستانی درباره COVID-19**

| | | | |
|---|---|---|---|
| تاریخ و ساعت: | C2 | شماره شرکت کننده: | C1 |
| شهر محل سکونت: | C4 | استان محل سکونت: | C3 |

والد گرامی، این پرسشنامه به‌منظور بررسی آگاهی، نگرش و عملکرد دانش آموزان دبیرستانی درباره COVID-19 طراحی شده‌است. خواهشمندیم در صورت تمایل فرزندتان و رضایت خودتان، سوالات را برای فرزندتان بخوانید و پاسخ های وی را **عیناً** در پرسشنامه علامت بزنید. لطفا در نظر داشته باشید که امانت‌داری شما در انتقال پاسخ‌های فرزندتان به ما در تعیین نیازهای آموزشی آن‌ها کمک می‌کند. همچنین، همه پاسخ‌ها محرمانه باقی می‌ماند و شما هر زمانی که بخواهید می توانید این مطالعه را ترک کنید.

| | |
|---|---|
| سن: ...... سال | C5 |
| جنس: زن ☐ مرد ☐ | C6 |
| قومیت: ۱. آذری/ترک ☐ ۲. فارس ☐ ۳. کرد ☐ ۴. لر ☐ ۵. سایر ☐ | C7 |
| مقطع تحصیلی: ۱. دبیرستان یک ☐ ۲. دبیرستان دو ☐ ۳. سایر ☐ (لطفا نام ببرید: ..........) | C8 |
| آیا کسی را می‌شناسید که مبتلا به COVID-19 شده باشد؟ ۱. بلی ☐ ۲. خیر ☐ | C9 |

| **لطفاً نظرتان را در مورد جملات زیر مشخص فرمائید (صحیح ☐ غلط ☐ نمی دانم ☐)** | |
|---|---|
| استفاده از خشک‌کن های دست به تنهایی نمی‌تواند موجب از بین رفتن عامل بیماری شود. | K1.1 |
| استفاده از واکسن آنفولانزا یا ذات‌الریه اثر حفاظتی علیه کرونا ندارد. | K1.2 |
| اعضای خانواده کرونا مشترک میان انسان و حیوان هستند و هر دو گروه را آلوده می‌کنند. | K1.3 |
| این بیماری همواره باعث ایجاد علائم خفیف می‌شود. | K1.4 |
| برای از بین بردن عامل بیماری می‌توان از بخور داغ استفاده کرد | K1.5 |
| برای از بین بردن عامل بیماری می‌توان با آب داغ استحمام کرد. | K1.6 |
| برای از بین بردن عامل بیماری می‌توان به سونا رفت. | K1.7 |
| برای از بین‌بردن عامل بیماری می‌توان داخل مجاری تنفسی را با سشوار گرم نگه داشت. | K1.8 |
| بیماری ناشی از کرونا در همه موارد مانند یک سرماخوردگی ساده است. | K1.9 |
| تمام بیماران مبتلا نیاز به بستری در بیمارستان و دریافت دارو دارند. | K1.10 |
| تمیز کردن دستان با محلول های ضدعفونی حاوی الکل از شستن آن ها با آب و صابون موثرتر است. | K1.11 |
| تنها در صورت وجود تب، بیمار ممکن است سایرین را نیز مبتلا کند. | K1.12 |
| خوردن سیر کرونا را از بین نمی‌برد. | K1.13 |
| در حال حاضر دارویی برای پیشگیری از این بیماری وجود ندارد. | K1.14 |
| در حال حاضر هیچ درمان اختصاصی برای این بیماری وجود ندارد. | K1.15 |

| | |
|---|---|
| K1.16 | در صورتی که فردی تب، سرفه یا تنگی نفس نداشته باشد نمی‌تواند حامل ویروس باشد. |
| K1.17 | شستشوی بینی با آب و نمک نقش حفاظتی علیه کرونا دارد. |
| K1.18 | شستشوی دهان با دهان‌شویه نقش حفاظتی علیه کرونا دارد. |
| K1.19 | شستن دست ها با آب برای از بین بردن عامل بیماری کافی نیست. |
| K1.20 | عامل این بیماری همان آنفولانزا است. |
| K1.21 | عامل این بیماری یک ویروس است. |
| K1.22 | عامل بیماری کرونا توسط حشرات منتقل نمی‌شود. |
| K1.23 | عامل کرونا از طریق قطرات تنفسی و نیز سطوح آلوده منتقل می‌شود. |
| K1.24 | فرد مبتلا به بیماری یا شخصی که تماس نزدیک با فرد مبتلا داشته‌اند باید حداقل ۱۴ روز قرنطینه شوند. |
| K1.25 | فردی که مبتلا به این بیماری بوده و اکنون خوب شده‌است، دیگر نیازی به رعایت نکایت بهداشتی و حفظ فاصله ایمنی ندارد. |
| K1.26 | کودکان به این بیماری مبتلا نمی‌شوند. |
| K1.27 | هر کس که به این بیماری مبتلا شود و خوب شود دیگر تا آخر عمر به آن مبتلا نمی‌شود. |
| K1.28 | هم‌سفر بودن با فرد ناقل عامل بیماری می‌تواند باعث انتقال آن شود. |

**کدام‌یک از موارد زیر می‌تواند از علائم COVID-19 باشد؟**

| | | | | |
|---|---|---|---|---|
| K2.1 | ازبین‌رفتن احساس بویایی یا چشایی | بلی ☐ | خیر ☐ | نمی دانم ☐ |
| K2.2 | اسهال | بلی ☐ | خیر ☐ | نمی دانم ☐ |
| K2.3 | آبریزش بینی | بلی ☐ | خیر ☐ | نمی دانم ☐ |
| K2.4 | بی‌اشتهایی | بلی ☐ | خیر ☐ | نمی دانم ☐ |
| K2.5 | تب | بلی ☐ | خیر ☐ | نمی دانم ☐ |
| K2.6 | تنگی نفس | بلی ☐ | خیر ☐ | نمی دانم ☐ |
| K2.7 | درد عضلانی | بلی ☐ | خیر ☐ | نمی دانم ☐ |
| K2.8 | سرفه | بلی ☐ | خیر ☐ | نمی دانم ☐ |
| K2.9 | ضعف و بی‌حالی | بلی ☐ | خیر ☐ | نمی دانم ☐ |
| K2.10 | عطسه | بلی ☐ | خیر ☐ | نمی دانم ☐ |
| K2.11 | گلودرد | بلی ☐ | خیر ☐ | نمی دانم ☐ |
| K2.12 | دیگر (لطفا ذکر کنید) ........................................................................................................ | | | |

**کدام یک از موارد زیر از جمله علائم خطر COVID-19 است؟**

| | | | | |
|---|---|---|---|---|
| K3.1 | تب طول کشیده بیشتر از پنج روز | بلی ☐ | خیر ☐ | نمی دانم ☐ |
| K3.2 | تشدید تنگی نفس | بلی ☐ | خیر ☐ | نمی دانم ☐ |
| K3.3 | تشدید سرفه‌ها | بلی ☐ | خیر ☐ | نمی دانم ☐ |
| K3.4 | کاهش سطح هوشیاری | بلی ☐ | خیر ☐ | نمی دانم ☐ |
| K3.5 | گیجی | بلی ☐ | خیر ☐ | نمی دانم ☐ |

| | |
|---|---|
| K3.6 | دیگر (لطفا ذکر کنید) ........................................................................................................... |
| T1 | آیا فکر می کنید که درباره COVID-19 به حد کافی اطلاعات دارید؟ بلی ☐ تا حدودی ☐ خیر ☐ |
| T2 | اطلاعات کنونی خود را در مورد COVID-19 از کجا کسب کرده اید؟ (می‌توانید بیش از یک مورد را علامت بزنید)<br>رادیو و تلویزیون ☐ تلگرام ☐ روزنامه و مجله ☐ پوستر و بروشور ☐ کادر درمان ☐<br>کلاس‌های آنلاین مدرسه ☐ اینستاگرام ☐ توییتر☐ خانوده و دوستان ☐ سایر موارد ☐<br>(لطفاً ذکر کنید): ........................................ |
| T3 | آیا احساس می کنید که در مورد COVID-19 نیاز به آموزش بیشتر دارید؟ بلی ☐ خیر ☐ |

| | |
|---|---|
| A1 | **در صورتی که خودتان به این بیماری مبتلا شوید، کدامیک از اقدامات زیر را انجام میدهید؟**<br>☐ مراجعه به پزشک در صورت تشدید علائم<br>☐ استراحت در منزل و ایزوله کردن خود در صورت علائم خفیف بیماری<br>☐ ادامه زندگی روزمره به شکل قبل |
| A2 | بنظر شما چقدر ممکن است که این ویروس توسط انسان ساخته شده باشد و بعنوان سلاح مورد استفاده قرار گرفته باشد؟ خیلی کم ☐ کم ☐ متوسط ☐ زیاد ☐ خیلی زیاد ☐ |
| A3 | وضعیت ایجاد شده فعلی را چقدر خطرناک ارزیابی میکنید؟ خیلی کم ☐ کم ☐<br>متوسط ☐ زیاد ☐ خیلی زیاد ☐ |
| A4 | بنظر شما ملت ها در برابر این بیماری پیروز خواهند شد؟ ☐ بلی ☐ خیر |
| A5 | فکر میکنید چقدر طول میکشد تا این بیماری کنترل شود؟<br>☐ کمتر از یک ماه<br>☐ یک تا سه ماه<br>☐ سه تا شش ماه<br>☐ شش تا نه ماه<br>☐ نه ماه تا یکسال<br>☐ بیش از یکسال<br>☐ نمیدانم |
| **لطفاً سؤالات زیر را بخوانید و پاسخ مورد نظرتان را با گذاشتن علامت × در داخل ☐ مشخص کنید**<br>(کاملاً موافقم ☐ موافقم ☐ مخالفم ☐ کاملاً مخالفم ☐) | |
| A6.1 | اگر یکی از نزدیکان بیمار شود حتما به عیادت وی خواهم رفت. |
| A6.2 | این روز ها ترجیح می‌دهم بجای مراجعه حضوری به مراکز خرید، از فروشگاه های اینترنتی خرید کنم. |
| A6.3 | با اینکه اصول رعایت فاصله ایمنی از سایر افراد را رعایت می‌کنم، ولی نسبت به دیدن افرادی که مبتلا به بیماری شده‌اند و اکنون خوب شده‌اند احساس خوبی ندارم. |

| | |
|---|---|
| با بروز اولین علائم مشکوک به بیماری به مسئولین مدرسه خود اطلاع می‌دهم. | A6.4 |
| با گرم شدن هوا همه‌گیری کرونا خودبخود خاتمه می‌یابد. | A6.5 |
| بستگان افراد متوفی در اثر این بیماری نباید احساس شرم کنند. | A6.6 |
| به غیر از گروه‌های پرخطر، گروه‌های دیگر ملزم به رعایت نکات بهداشتی پیشگیرانه نیستند. | A6.7 |
| تعطیل شدن مدارس بدلیل همه‌گیری کرونا فرصت مناسبی است تا با دوستان و خانواده دیدار حضوری داشته باشم. | A6.8 |
| حفظ فاصله اجتماعی و رعایت توصیه های بهداشتی وظیفه هر شهروند در این همه‌گیری است. | A6.9 |
| در خانه هر کس باید حوله شخصی خودش را داشته باشد یا از دستمال کاغذی برای خشک کردن دستان استفاده کند. | A6.10 |
| صاحبان حیوانات خانگی باید مراقب تماس‌های خود و سایرین با حیوانشان باشند. | A6.11 |
| مسئولین باید بجای توجه به این بیماری به مشکلات بزرگتر مانند تصادفات جاده‌ای و بیماری‌های غیرواگیر که تعداد افراد بیشتری را درگیر می‌کند توجه کنند. | A6.12 |
| هر فردی که سابقه سفر به مناطق آلوده و یا سابقه تماس نزدیک با فرد مشکوک به بیماری را داشته باشد باید به وزارت بهداشت اطلاع دهد. | A6.13 |
| همه شهرهای درگیر باید بلافاصله قرنطینه کامل شوند. | A6.14 |
| همه‌گیری این بیماری در سطح جهانی ناشی از گناهان انسان ها و انتقام خداوند است. | A6.15 |

| | |
|---|---|
| آیا خودتان به این بیماری مبتلا شده اید؟<br>☐ بلی، تستم مثبت شده بود. ☐ خیر ☐ برخی علائم مشکوک را داشتم ولی اقدامی نکردم. | P1 |
| **برای پیشگیری از بیماری کدامیک از اقدامات زیر را انجام داده‌اید؟**<br>☐ ماندن در خانه<br>☐ رعایت فاصله حداقل دومتری از سایرین<br>☐ رعایت بهداشت دستان<br>☐ استفاده از ماسک<br>☐ استفاده از دستکش<br>☐ استفاده از مکمل‌های ویتامین<br>☐ مصرف جوشانده گیاهی<br>☐ حجامت<br>☐ داروی امام کاظم<br>☐ هیچکدام<br>☐ سایر (لطفا ذکر کنید) | P2 |
| در صورت استفاده از ماسک، آن را چند ساعت استفاده میکنید؟ | P3 |

| | | |
|---|---|---|
| P4 | | از ابتدای شروع همه‌گیری، بیشتر از چه نوع ماسکی جهت پوشاندن دهان و بینی خود استفاده کرده‌اید و می‌کنید؟<br>☐ ماسک استفاده نکرده و نمی‌کنم<br>☐ ماسک جراحی<br>☐ ماسک N95<br>☐ ماسک پارچه‌ای<br>☐ ماسک دست‌ساز خودم یا یکی از اعضای خانواده<br>☐ سایر |
| P5 | | در هفته اخیر چند بار از خانه خارج شده اید؟ |
| P6 | | هدف شما از خروج از منزل چه بوده است؟<br>☐ از منزل خارج نشدم<br>☐ خرید مایحتاج روزانه<br>☐ خرید اقلام غیر از خوار و بار<br>☐ دیدار اقوام و دوستان<br>☐ گردش<br>☐ مراجعه به مراکز درمانی<br>☐ سایر (لطفا ذکر کنید) |
| P7 | | در روز چند مرتبه دستان خود را با آب و صابون می‌شویید؟ |
| P8 | | در هر نوبت، چند ثانیه دستان خود را با آب و صابون می‌شویید؟ |
| P9 | | در روز چند مرتبه دستان خود را با محلول های ضدعفونی کننده حاوی الکل پاک می‌کنید؟ |
| P10 | | در هر نوبت، چند ثانیه دستان خود را با محلول های ضد عفونی کننده حاوی الکل پاک می‌کنید؟ |
| | | **در زمان وقوع همه گیری، کدام یک از اقدامات زیر را انجام داده اید؟** |
| P11.1 | ☐ | انواعی دارو جهت پیشگیری از این بیماری تهیه کرده‌ام. |
| P11.2 | ☐ | تعدادی ماسک برای حفاظت فردی خریداری کرده‌ام. |
| P11.3 | ☐ | در خانه برای خودم یا خانواده ام ماسک دست‌ساز درست کرده‌ام. |
| P11.4 | ☐ | در خارج از منزل، محلول ضد عفونی کننده دست یا محلول آب و صابون با خود همراه دارم. |
| P11.5 | ☐ | در صورت فوت آشنایان در اثر این بیماری در مراسم تشییع شرکت کردم. |
| P11.6 | ☐ | در پنجشنبه آخر سال به زیارت اهل قبور رفتم. |
| P11.7 | ☐ | در روز های پایانی سال منتهی به ایام نوروز برای خرید به بازار رفتم. |
| P11.8 | ☐ | پس از ورود به منزل با خرید، بسته بندی اقلام خریداری شده را با آب و صابون یا ماده ضد عفونی کننده دیگر می‌شویم. |
| P11.9 | ☐ | نان را قبل از استفاده برای از بین رفتن عامل بیماری گرم می‌کنم. |
| P11.10 | ☐ | در خانه سطوحی که زیاد لمس میشوند مانند کنترل تلویزیون، دستگیره درب، کلید و پریز، سطح میز ها، کیبورد و ماوس را مرتبا ضد عفونی می‌کنم. |

| | | |
|---|---|---|
| P11.11 | □ | در صورتی که بیرون از منزل باشم، تلفن همراهم را از جیبم خارج نمی‌کنم تا آلوده نشود. |
| P11.12 | □ | تلفن همراهم را مرتبا طبق راهنما ضدعفونی می‌کنم. |
| P11.13 | □ | امسال دید و بازدید نوروزی را به رسم هر سال انجام دادم. |
| P11.14 | □ | امسال دید و بازدید نوروزی را به رسم هر سال انجام خواهم داد. |
| P11.15 | □ | در صورتی که یکی از علائم تب، سرفه یا تنگی نفس را داشته باشم خود را در خانه قرنطینه می‌کنم. |
| P11.16 | □ | بمحض توصیه مقامات بهداشتی به خانه نشینی، قرنطینه را در منزل شروع کردم. |
| P11.17 | □ | در صورتی که بنابر ضرورت مجبور به ترک منزل شوم حتما از ماسک استفاده می‌کنم. |
| P11.18 | □ | پیش از دست زدن به صورت خود دست های خود را با آب و صابون می شویم یا با الکل ضدعفونی می‌کنم. |
| P11.19 | □ | هنگام عطسه یا سرفه کردن، دهان و بینی ام را با یک دستمال می‌پوشانم. |
| P11.20 | □ | امسال در ایام نوروز مسافرت نرفتم. |
| P11.21 | □ | امسال در ایام نوروز مسافرت نخواهم رفت. |
